# Evaluation of the efficacy of low-level laser in improving the symptoms of burning mouth syndrome

**DOI:** 10.4317/jced.52298

**Published:** 2015-10-01

**Authors:** Fateme Arbabi-Kalati, Nour-Mohammad Bakhshani, Maryam Rasti

**Affiliations:** 1 Oral Medicine Department, Zahedan University of Medical Sciences, Zahedan, Iran; 2 Oral and Dental Disease Research Center, Zahedan University of Medical Sciences; 3 Research Center for Children and Adolescent’s Health, Baharan psychiatric center Zahedan University of Medical Sciences, Zahedan, Iran; 4 Dentist

## Abstract

**Background:**

Burning mouth syndrome (BMS) is common conditions that affects menopause women, patients suffer from sever burning sensation. Up to now there is no definitive treatment for this disease. Present study was undertaken to evaluate the efficacy of low-level laser (LLL) in improving the symptoms of burning mouth syndrome.

**Material and Methods:**

Twenty patients with BMS were enrolled in this study; they were divided in two groups randomly. In the laser group, in each patient, 10 areas on the oral mucosa were selected and underwent LLL irradiation at a wavelength of 630 nm, and a power of 30 mW for 10 seconds twice a week for 4 weeks. In the placebo group, silent/off laser therapy was carried out during the same period in the same areas. Burning sensation and quality of life were evaluated.

**Results:**

Burning sensation severity and quality of life in the two groups after intervention were different significant statistically, (*p*= 0.004, *p*= 0.01 respectively) .Patients in laser group had better results.

**Conclusions:**

It can be concluded that low level laser might decrease the intensity of burning mouth syndrome.

** Key words:**Pain, low-level laser, burning mouth syndrome, oral mucosa.

## Introduction

Burning mouth syndrome (BMS) is a common condition, with symptoms such as pain and a burning sensation of the oral mucosa in the absence of manifestations of any local condition. The condition predominantly affects the tongue, especially the anterior two-thirds of the tongue (1,2). However, it can affect other areas of the oral cavity, too, including the lips, the buccal mucosa, the floor of the mouth, the gingiva and the hard palate (1). The symptoms vary from mild to severe in intensity. Patients complain of xerostomia, lack of or absence of taste or altered taste sensation (1). Despite a large number of studies, the true nature of BMS is still controversial and is shrouded in mystery and there is no universal consensus on the diagnosis, etiology and treatment of BMS (2). International Association for the Study of Pain defines this condition as “burning pain in the tongue or other oral mucous membrane associated with normal signs and laboratory findings lasting at least 4-6 months” (3).

It appears the prevalence of BMS is higher in middle-aged women and after menopause. The female-to-male ratio has been reported from 3:1 to 16:1 (4). The patients usually wake up in the morning without any pain and the symptoms increase in severity during the day until the afternoon. Many patients suffer from this condition for a long time, persisting for many months or years (4).

The treatment of BMS is usually symptomatic and use of low doses of benzodiazepines, clonazepam and tricyclic antidepressants might be effective (5,6). However, long-term treatment with such medications might result in xerostomia, aggravating the patients’ pain perception (1,4). A number of researchers have reported a definite decrease in pain severity with the application of low-level laser beams and have shown that it can decrease the severity of pain in stomatitis and BMS.

Considering the small sample sizes in the studies above and use of laser beams with varying wavelengths and energy levels and the discrepancies between the results of some studies, the present study was undertaken to evaluate the efficacy of low-level laser in improving the symptoms of burning mouth syndrome. Since none of the studies above have evaluated the quality of life of such patients, in the present study the general quality of life of these patients was evaluated, too.

## Material and Methods

The present clinical trial was approved by the Ethics Committee of Zahedan University of Medical Sciences and was registered at the website of Iranian Clinical Trials at www.irct.ir under the code IRCT201312173133N7.

All the patients signed an informed consent form before being included in the study. The subjects were selected from the patients with burning mouth syndrome, who referred to Zahedan Faculty of Dentistry. The criteria used to diagnose BMS consisted of the following: 1) burning sensation in all or a part of the oral cavity with or without symptoms such as a change in taste sensation for at least 4 months; 2) normal oral mucosa without any lesion; and 3) absence of any local or systemic factors which produce the same symptoms.

Exclusion criteria consisted of any known systemic condition, patients under 18, pregnancy, smoking, patients with oral lesions and patients not signing the informed consent form. The patients were examined before the study and the severity of burning sensation was evaluated based on numeric rating scale (NRS), in which zero indicated absence of burning sensation and a score of 10 indicated the most severe burning sensation experienced by the patient. Each patient was asked to rate his/her burning sensation severity from zero to 10. The patients were divided into laser and placebo groups using block randomization.

In the laser group, in each patient, 10 areas on the oral mucosa, 2 areas on the buccal mucosa on each side, 2 areas on the tongue, 2 areas on the floor of the mouth, 1 area on the soft palate and 1 area on the hard palate were selected and underwent LLL irradiation at a wavelength of 630 nm, and a power of 30 mW for 10 seconds twice a week for 2 weeks. To this end, Iodi-ne-Gallium-Aresnide laser of Mustange laser device (Russia) was used. The laser dose for each area was 1 j/cm2.

In the placebo group, silent/off laser therapy was carried out during the same period in the same areas. All the patients wore protective glasses to protect their eyes against laser injuries and to blind the patients to the type of treatment modality used.

All the patients were examined by an oral medicine specialist before laser irradiations and their pain severity was recorded. In addition, a questionnaire was completed for each patient for quality of life. The validity and reproducibility of the Persian version of the questionnaire have been evaluated and confirmed by Motallebenjad *et al.* (7).

Each patient was treated for 2 weeks. At the end of the treatment period, all the patients were examined once again and their pain severity was recorded. Then the questionnaire for quality of life was completed again. All evaluations of pain and quality of life (before and after treatment) were done by blind oral medicine specialist.

Data were analyzed with SPSS 18. The means of burning sensation severity scores of the patients before and after intervention were evaluated by Mann-Whitney U test. Statistical significance was defined at *P*<0.05.

## Results

Twenty female patients (10 in the laser group and 10 in the placebo group) were included in the present study, age and duration of disease are showed in  Table 1; there was no statistically significant differences between two groups.  Table 2 presents the scores of burning sensation severity and quality of life in the two groups before and after intervention. As  Table 2 have been shown there were no statistically significant differences between two groups before intervention but after intervention burning sensation severity was lower and quality of life was better in laser group, there were statistically significant differences between two groups. (*P*= 0.004, *P*= 0.01 respectively).

**Table 1:**
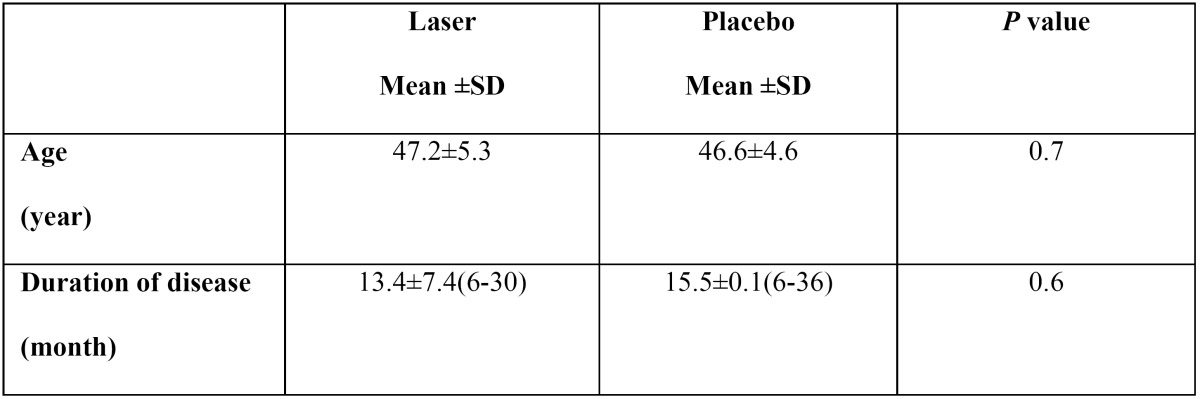
Demographic characteristics of patients.

**Table 2:**
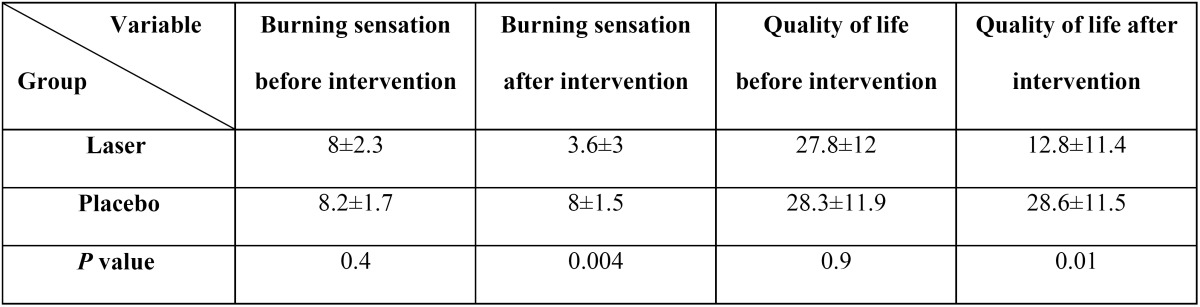
The severities of burning sensation and quality of life in the two groups before and after treatment.

## Discussion

The results of the present study showed that use of low-level laser (LLL) in patients with BMS results in a decrease in pain severity, though it does not relieve pain completely.

A limited number of studies have evaluated the effect of LLL on BMS and almost all these studies have reported a decrease in burning sensation severity after treatment, consistent with the results of the present study (4,8,9).

In a pilot study by Romeo *et al.* (4) in 2010 the effect of LLL was evaluated on 25 patients with BMS. Patients were irradiated with double diode laser 650 nm and 910 nm, with a fluence of 0.53 J/cm for 15 minutes twice a week for 4 weeks. All the patients exhibited a decrease in burning sensation severity and all of them attributed it to the effect of LLL; 68% of patients exhibited a real decrease in pain severity and pain did not aggravate in any of the patients.

In another study by Kato *et al.* (8) in 2010, 11 patients were irradiated with infrared laser with a wavelength of 790 nm for 3 consecutive weeks; 84% of the patients reported improvements after receiving LLL .

In 2011, Yang and Huang (10) made an effort to treat BMS with LLL. Seventeen patients were irradiated 1-7 times with 800-nm wavelength diode laser and average power of 1.5 W/cm2; there was a 46.7% decrease in pain severity .

In another study in 2011, Dos Santos (9) evaluated the effect of LLL; A continuous wavelength of 660 nm, power 0.8 J/point on BMS for 10 weeks. All 10 the patients recovered from the condition and there was a 58% decrease in pain severity .

Pezelj-Ribarić *et al.* (1) in 2012 determined the salivary levels of proinflammatory cytokines in patients with BMS before and after LLL irradiation and reported a significant decrease in unstimulated salivary levels of two proinflammatory factors of TNF-α and IL-6 four weeks after treatment with laser; however, the patients’ burning sensation did not decrease.

The mechanism of the effect of LLL on decreasing pain and burning sensation severity is unknown; however, some studies have shown that there are three main mechanisms for the analgesic effects of LLL, including induction of the release of endogenous opiates, an increase in pain threshold and regulation of the release of pain mediators such as bradykinin and histamine (11). In addition, some studies have shown that LLL irradiation changes the rate of action potential conductance along the nerves. LLL retards pain impulse conductance through the autonomous nervous system, regulation of serotonin and norepinephrine levels and by increasing the pain threshold.

The results of the present study show that using LLL significantly decrease burning sensation severity in patients who suffer from BMS, but the present study had some limitations, including the small sample size and long duration of the application of LLL, which decreases the cooperation of patients. Further research is required to validate our findings.
